# Monitoring Troponin I and CRP During Clozapine Titration: Assessing Adherence to Myocarditis Protocols

**DOI:** 10.1192/bjo.2026.11429

**Published:** 2026-06-30

**Authors:** Tasfique Chowdhury, Alistair Martin

**Affiliations:** Rotherham Doncaster and South Humber NHS Foundation Trust, Rotherham, United Kingdom

## Abstract

**Aims:**

Clozapine remains the only licensed treatment for patients with treatment-resistant schizophrenia and is highly effective, but its use is limited by potentially serious adverse effects such as clozapine-associated myocarditis, as has been highlighted by the recent position statement by the RCPsych. The risk of myocarditis is greatest within the first 6–8 weeks of treatment, during which early detection relies on monitoring inflammatory markers and cardiac biomarkers, particularly CRP and troponin.

Evidence-based monitoring for myocarditis is guided by the Ronaldson et al. algorithm, which is adopted by the Maudsley Prescribing Guidelines and supported by ZTAS, recommending weekly troponin and CRP monitoring during the first four weeks of titration. However, under local protocols normal reference ranges are often based on acute cardiac pathways, meaning that mildly raised troponin levels often prompt additional testing, leading to unnecessary investigations and resource use.

This audit evaluated adherence to guideline-recommended monitoring of troponin and CRP during clozapine initiation or re-titration on inpatient general adult psychiatry wards. It also assessed the management of abnormal results and examined whether troponin testing was over-utilised when not clinically indicated.

**Methods:**

A retrospective audit was conducted at Swallownest Court (RDaSH NHS Foundation Trust), reviewing all patients who commenced or retitrated clozapine between 1 July and 30 November 2025. Data were extracted from electronic records, including baseline investigations, weekly troponin and CRP results, and clinical responses to abnormal findings. Practice was compared against Maudsley and Ronaldson monitoring standards.

**Results:**

Ten patients were included. Baseline troponin and CRP were documented in 80% and baseline ECG in 90%. Five patients (50%) developed mildly elevated troponin levels (>3 ng/L, but <13ng/L), none of which exceeded myocarditis-specific thresholds (greater than 2×ULN) or were associated with clinical symptoms. Nevertheless, 80% of these cases underwent repeat troponin testing. CRP elevations occurred in four patients; two were attributed to non-cardiac causes. No cases of suspected myocarditis were identified. Findings indicate that repeat troponin tests were frequently triggered by ACS-based reference values rather than guideline-recommended myocarditis thresholds.

**Conclusion:**

Monitoring during clozapine titration is generally well conducted; however, troponin testing is commonly repeated in response to mild, clinically insignificant elevations, leading to unnecessary investigations. Recommendations have been made to align local practice with the Ronaldson and Maudsley protocols, incorporating locally agreedbiochemistry reference ranges. These will be implemented following inclusion in local policy, with re-audit planned to assess their impact on monitoring practice and patient safety.

